# Hemoglobin A1c Levels and Risk of Severe Hypoglycemia in Children and Young Adults with Type 1 Diabetes from Germany and Austria: A Trend Analysis in a Cohort of 37,539 Patients between 1995 and 2012

**DOI:** 10.1371/journal.pmed.1001742

**Published:** 2014-10-07

**Authors:** Beate Karges, Joachim Rosenbauer, Thomas Kapellen, Verena M. Wagner, Edith Schober, Wolfram Karges, Reinhard W. Holl

**Affiliations:** 1 Division of Endocrinology and Diabetes, RWTH Aachen University, Aachen, Germany; 2 Institute for Biometrics and Epidemiology, German Diabetes Center, Leibniz Center at University of Düsseldorf, Düsseldorf, Germany; 3 Department of Pediatrics, University of Leipzig, Leipzig, Germany; 4 Department of Pediatrics, University of Lübeck, Lübeck, Germany; 5 Department of Pediatrics, Medical University of Vienna, Vienna, Austria; 6 Institute of Epidemiology and Medical Biometry, University of Ulm, Ulm, Germany; University of Oxford, United Kingdom

## Abstract

**Background:**

Severe hypoglycemia is a major complication of insulin treatment in patients with type 1 diabetes, limiting full realization of glycemic control. It has been shown in the past that low levels of hemoglobin A1c (HbA1c), a marker of average plasma glucose, predict a high risk of severe hypoglycemia, but it is uncertain whether this association still exists. Based on advances in diabetes technology and pharmacotherapy, we hypothesized that the inverse association between severe hypoglycemia and HbA1c has decreased in recent years.

**Methods and Findings:**

We analyzed data of 37,539 patients with type 1 diabetes (mean age ± standard deviation 14.4±3.8 y, range 1–20 y) from the DPV (Diabetes Patienten Verlaufsdokumentation) Initiative diabetes cohort prospectively documented between January 1, 1995, and December 31, 2012. The DPV cohort covers an estimated proportion of >80% of all pediatric diabetes patients in Germany and Austria. Associations of severe hypoglycemia, hypoglycemic coma, and HbA1c levels were assessed by multivariable regression analysis. From 1995 to 2012, the relative risk (RR) for severe hypoglycemia and coma per 1% HbA1c decrease declined from 1.28 (95% CI 1.19–1.37) to 1.05 (1.00–1.09) and from 1.39 (1.23–1.56) to 1.01 (0.93–1.10), respectively, corresponding to a risk reduction of 1.2% (95% CI 0.6–1.7, *p*<0.001) and 1.9% (0.8–2.9, *p*<0.001) each year, respectively. Risk reduction of severe hypoglycemia and coma was strongest in patients with HbA1c levels of 6.0%–6.9% (RR 0.96 and 0.90 each year) and 7.0%–7.9% (RR 0.96 and 0.89 each year). From 1995 to 2012, glucose monitoring frequency and the use of insulin analogs and insulin pumps increased (*p*<0.001). Our study was not designed to investigate the effects of different treatment modalities on hypoglycemia risk. Limitations are that associations between diabetes education and physical activity and severe hypoglycemia were not addressed in this study.

**Conclusions:**

The previously strong association of low HbA1c with severe hypoglycemia and coma in young individuals with type 1 diabetes has substantially decreased in the last decade, allowing achievement of near-normal glycemic control in these patients.

*Please see later in the article for the Editors' Summary*

## Abstract 2

In a cohort study, Beate Karges and colleagues find that the association between low hemoglobin A1C and severe hypoglycemia in children and young adults with type 1 diabetes has decreased over the period between 1995 and 2012.

*Please see later in the article for the Editors' Summary*

## Editors' Summary

**Background:**

Worldwide, more than 380 million people have diabetes, a chronic disorder characterized by high levels of glucose (sugar) in the blood. Blood sugar levels are usually controlled by insulin, a hormone produced by the pancreas. In people with diabetes, blood sugar control fails because they make no insulin (type 1 diabetes) or because the cells that normally respond to insulin by removing sugar from the blood have become insulin-resistant (type 2 diabetes). Type 1 diabetes, which tends to develop in childhood or early adulthood, is responsible for about 10% of cases of diabetes in adults and is treated with injections of insulin. Type 2 diabetes can usually be treated with diet, exercise, and antidiabetic drugs. With both types of diabetes, it is important to keep blood sugar levels within the normal range (good glycemic control) to reduce the long-term complications of diabetes, which include kidney failure, blindness, and an increased risk of cardiovascular disease.

**Why Was This Study Done?:**

Patients with type 1 diabetes can achieve strict glycemic control using intensive insulin therapy, but such treatment is associated with a risk of severe or fatal hypoglycemia (low blood sugar). Past studies have found an association between low levels of hemoglobin A1c (HbA1c, a marker of average blood sugar levels over the past 2–3 months; a low HbA1c percentage indicates good glycemic control) and a high risk of severe hypoglycemia. Because of this inverse association, people at risk of severe hypoglycemia are advised to aim for an HbA1c of 7.5% or less, which puts them at risk of diabetic complications (most adults with diabetes aim for an HbA1c of 6.5% or less; people without diabetes have Hb1Ac readings below 6.05%). With recent improvements in insulin therapy, it is not clear whether the inverse association between the incidence of severe hypoglycemia and HbA1c levels still exists. In this trend analysis, the researchers investigate the association over time between HbA1C levels and the risk of severe hypoglycemia in a large cohort (group) of Austrian and German children and young adults with type 1 diabetes.

**What Did the Researchers Do and Find?:**

The researchers analyzed data on Hb1Ac levels and on incidents of severe hypoglycemia and hypoglycemic coma collected from 37,539 children and young adults with type 1 diabetes between 1995 and 2012 by the DPV (Diabetes Patienten Verlaufsdokumentation) Initiative for diabetes care. The DPV cohort includes around 80% of all children and young adults with type 1 diabetes in Germany and Austria. Over the study period, the use of insulin analogs (compounds related to insulin that keep blood sugar levels steadier than regular insulin injections) and of insulin pumps (which deliver constant amounts of short-acting insulin analogs to the body) increased, and there was an increase in how often patients monitored their blood sugar level. Notably, between 1995 and 2012, the relative risk for severe hypoglycemia per 1% decrease in Hb1Ac declined from 1.28 to 1.05, and the relative risk for hypoglycemic coma per 1% decrease in Hb1Ac declined from 1.39 to 1.01. That is, the strength of the inverse association between severe hypoglycemia or coma and HbA1c decreased during the study period. Expressed another way, between 1995 and 2012, the relative risk for severe hypoglycemia and coma per 1% HbA1c decrease dropped by 1.2% and 1.9%, respectively, each year.

**What Do These Findings Mean?:**

These findings reveal a substantial decrease since 1995 in the previously strong inverse association between low HbA1c levels and severe hypoglycemia and hypoglycemic coma in this cohort of young Germans and Austrians with type 1 diabetes. This decrease mainly occurred because of substantial reductions in the risk of hypoglycemia in patients with HbA1c levels between 6.0% and 7.9%, but the study provides no information about the drivers of this reduction. Moreover, these findings may apply only to young type 1 diabetes patients of European descent, and their accuracy may be limited by other aspects of the study design. However, by showing that HbA1c has become a minor predictor for severe hypoglycemia in this group of patients, these findings suggest that strict glycemic control in young patients with type 1 diabetes has become safer in recent years. Thus, it should now be possible to reduce the risk of long-term diabetic complications in such patients through achievement of near-normal glycemic control without increasing patients' risk of severe hypoglycemia.

**Additional Information:**

Please access these websites via the online version of this summary at http://dx.doi.org/10.1371/journal.pmed.1001742.

- The US National Diabetes Information Clearinghouse provides information about diabetes for patients, health care professionals, and the general public (in English and Spanish), including information on the HbA1c test and a description of a trial that compared the effects of intensive versus conventional treatment of blood glucose levels on the development of diabetic complications in patients with type 1 diabetes
- The UK National Health Service Choices website provides information for patients and carers about type 1 diabetes, including a video that describes parents' experiences caring for a child with type 1 diabetes, and information about treating type 1 diabetes that includes a short video about HbA1c
- The charity Diabetes UK provides detailed information about type 1 diabetes for patients and carers
- The UK-based non-profit organization Healthtalkonline provides information about type 1 diabetes and young people, including interviews with young people about their experiences of the condition
- MedlinePlus provides links to further resources and advice about type 1 diabetes (in English and Spanish)
- Information about the DPV Initiative is available (mainly in German)

## Introduction

Maintenance of near-normal glycemic control is a central goal for individuals with diabetes to reduce diabetic complications of the eye, kidney, nerves, and cardiovascular system [1],[2]. Strict metabolic control can be achieved by intensive insulin therapy in patients with type 1 diabetes, which is associated with the risk of severe hypoglycemia [1]–[6]. Since hypoglycemia is a major complication in patients with type 1 diabetes [2],[7],[8], accounting for up to 6% to 7% of disease-related mortality [9],[10], several strategies have been developed to predict and prevent hypoglycemia [11]–[14].

A strong inverse association between the incidence of severe hypoglycemia and hemoglobin A1c (HbA1c) levels has been described in previous decades in adults [1] and children [4],[5],[15] with type 1 diabetes using intensive insulin therapy. Later analyses confirmed HbA1c as a main predictor for severe hypoglycemia in children with diabetes [6],[11],[16], while more recent reports found no association between the incidence of severe hypoglycemia and HbA1c levels [17]–[20]. A few studies have evaluated temporal trends of severe hypoglycemia and HbA1c [8],[16],[17],[21], but the direct association between HbA1c and severe hypoglycemia or hypoglycemic coma over time has not been studied in depth in large populations of patients with type 1 diabetes.

The aim of this study was to investigate temporal trends in the association between severe hypoglycemia (defined as an event requiring assistance of another person) and HbA1c levels in a large cohort of young patients with type 1 diabetes. We have previously shown that the use of insulin analogs and continuous subcutaneous insulin infusion (insulin pump) has increased during the past decade in these patients [8]. At the same time, a reduction of severe hypoglycemic events has been observed while metabolic control has been maintained or improved. Based on these findings, we hypothesized that the inverse association between severe hypoglycemia and HbA1c has substantially weakened in recent years. If confirmed, lower HbA1c would no longer predict severe hypoglycemia, thereby reducing barriers to achieving near-normal blood glucose in type 1 diabetes patients.

## Methods

### Ethics Statement

Informed consent for participation in the DPV Initiative was obtained from patients or their parents by verbal or written procedure, as approved by the responsible commissioners for data protection of each participating center. Analysis of anonymized data within the DPV Initiative was approved by the Ethics Committee of Ulm University, Ulm, Germany.

### Study Design

The DPV (Diabetes Patienten Verlaufsdokumentation) Initiative in Germany and Austria has prospectively followed patients with diabetes mellitus since January 1, 1995 [8],[22],[23]. As of December 31, 2012, 372 diabetes centers (hospitals and practices) have documented the treatment and outcomes of routine diabetes care using the DPV software as previously described in detail [8]. The DPV database covers an estimated proportion of >80% of all pediatric diabetes patients in Germany and Austria. In this study, inclusion criteria were the clinical diagnosis of type 1 diabetes; age 1 to 20 y; regular documentation of HbA1c, insulin therapy, and hypoglycemia; and treatment between 1995 and 2012, resulting in *n* = 53,474 eligible patients. Exclusion criteria were diabetes duration less than 2 y (*n* = 11,246), HbA1c <6% (<42 mmol/mol) (*n* = 715), comorbid celiac disease (*n* = 1,397), and place of birth of one or both parents outside Germany or Austria (*n* = 2,577). For each patient, clinical data (HbA1c, body mass index [BMI], number of injections per day, daily insulin dose, and frequency of self-monitoring of blood glucose [SMBG]) were averaged per year of follow-up, and numbers of hypoglycemic events were added up. Thus, the analysis dataset originated from independent cross-sectional cohorts of patients by calendar year.

### Study Parameters

Severe hypoglycemia was defined according to the American Diabetes Association Workgroup on Hypoglycemia [24] as an event requiring assistance of another person to actively administer carbohydrates, glucagon, or intravenous glucose. Hypoglycemic coma was defined as loss of consciousness or occurrence of seizures, consistent with previous classifications [25],[26]. In preschool children, severe hypoglycemia was defined as the presence of altered mental status and the inability to assist in their care, and coma as unconsciousness or occurrence of convulsions, requiring parenteral therapy [25],[26]. Hypoglycemic events and other parameters were actively enquired about and documented at each medical visit using the standardized DPV questionnaire [8].

In order to adjust for different laboratory methods (variation of normal HbA1c, mean ± standard deviation [SD] 5.4%±0.28%, median 5.3%, quartile 1 [Q1] 5.0%, quartile 3 [Q3] 5.4%), local HbA1c values were mathematically standardized to the Diabetes Control and Complications Trial (DCCT) reference range 4.05%–6.05% using the multiple-of-the-mean transformation method [1],[8] by multiplying the ratio of the individual HbA1c value to the normal mean HbA1c value of the respective local laboratory by the normal mean of the DCCT (5.05%). BMI values were transformed to standard deviation scores based on reference values by applying the LMS method as described before [8],[27].

### Statistical Analyses

Rates of severe hypoglycemia and coma were expressed per 100 patient-years with 95% CIs based on normal approximation of the Poisson distribution. For descriptive analysis, mean and SD were calculated for continuous variables, and percentages for categorical variables. Mean rates of severe hypoglycemia and coma were compared between years of treatment based on a Poisson distribution of events.

To assess the effect of potentially influencing factors on the rate of severe hypoglycemia and hypoglycemic coma, multivariable negative binominal regression analysis was conducted in order to account for over-dispersion of events. To analyze trends in the rate of severe hypoglycemia, models including year of treatment as an independent continuous term were fitted, with adjustments for HbA1c, sex, age, diabetes duration, and insulin treatment regimen (1–3 or ≥4 insulin injection time points per day, or insulin pump therapy). To assess the association between HbA1c and the rate of severe hypoglycemia, models including HbA1c as a continuous term were fitted, with adjustments for sex, age, diabetes duration, and insulin treatment regimen. To investigate trends in the associations between HbA1c, sex, age, and diabetes duration and the rate of severe hypoglycemia, the year of treatment and terms for interaction between these variables and year of treatment were included in the regression models. In additional analyses, HbA1c (6.0%–6.9% [42–52 mmol/mol], 7.0%–7.9% [53–63 mmol/mol], 8.0%–8.9% [64–74 mmol/mol], or ≥9.0% [≥75 mmol/mol]) was modeled as a categorical effect. Results of regression analyses are presented as mean adjusted rates or relative risks (RRs) including 95% CIs. Wald tests were used for hypothesis testing. 

*p*-Values of two-sided tests <0.05 were considered statistically significant. All analyses were performed with SAS for Windows, version 9.3 (SAS Institute).

## Results

### Study Population

The entire study population consisted of 37,539 individuals (17,793 females, 19,746 males) with a mean age ± SD of 14.4±3.8 y from 349 diabetes centers (listed in Text S1). Clinical characteristics of the study population are described in Table 1. Mean age of patients declined from 17.0±2.9 y in 1995 to 13.4±3.7 y in 2012, with an average annual decrease of 0.094±0.004 y (Wald test for trend: *p*<0.001). Diabetes duration decreased from 8.1±4.2 y in 1995 to 5.9±2.8 y in 2012, with an average annual decrease of 0.084±0.003 y (Wald test for trend: *p*<0.001). There was no temporal trend in the sex distribution of the study population from 1995 to 2012 (annual change in proportion of males 0.066%±0.052%, Wald test for trend: *p* = 0.21).

**Table 1:** Characteristics of patients with type 1 diabetes treated between 1995 and 2012.

Characteristic	Result
**Number of patients**	37,539
**Age, ** ***n*** ** (percent)**	
1 to 5.9 y	981 (2.6%)
6 to 11.9 y	8,637 (23.0%)
12 to 20 y	27,921 (74.4%)
**Male sex, percent**	52.6%
**Mean age ± SD, years**	14.4±3.8
**Mean diabetes duration ± SD, years**	6.2±3.2
**Mean HbA1c ± SD, percent (mmol/mol)** *	8.3%±1.6% (67±13)
**BMI standard deviation score (** ***n*** ** = 36,694)**	0.6±0.9
**Insulin treatment regimen, ** ***n*** ** (percent)**	
1–3 insulin injection time points per day	2,914 (7.8%)
≥4 insulin injection time points per day	24,027 (64.0%)
Insulin pump therapy	10,598 (28.2%)
**Total daily insulin dose per kilogram (** ***n*** ** = 37,028)**	0.89±0.29
**Prandial/total insulin ratio (** ***n*** ** = 37,239)**	0.6±0.2
**Use of short-acting analogs, ** ***n*** ** (percent)**	20,248 (54.0%)
**Use of long-acting analogs, ** ***n*** ** (percent)**	12,955 (34.5%)
**SMBG frequency per day (** ***n*** ** = 34,558)**	4.9±1.8

*DCCT reference range 4.05%–6.05%.

The average number of HbA1c measurements per patient per year was 3.2±1.8. In total, 118,811 HbA1c measurements were available for analysis. Mean HbA1c decreased from 8.9%±1.8% (74±15 mmol/mol) in 1995 to 8.0%±1.3% (64±13 mmol/mol) in 2012, with an average annual decrease of 0.036%±0.002% (Wald test for trend: *p*<0.001). The percentage of individuals using insulin pumps continuously rose from 1.3% in 1995 to 47% in 2012, with an average absolute annual increase of 3.2%±0.04% (Wald test for trend: *p*<0.001). Total daily insulin dose per kilogram showed a very small but significant increase over time (annual change in daily insulin dose per kilogram 0.0006±0.0003 units, Wald test for trend: *p* = 0.049). Mean prandial-to-total-insulin ratio was 0.5±0.2 in each year before 2003 and 0.6±0.2 in each year after 2004 (annual change in prandial-to-total-insulin ratio 0.0047±0.0002, Wald test for trend: *p*<0.001).

The proportion of patients using short-acting insulin analogs continuously increased from 4.3% in 1996 to 74.6% in 2012, with an average annual increase of 4.3%±0.04% (Wald test for trend: *p*<0.001). The proportion of individuals using long-acting insulin analogs increased from 1.3% in 2000 to 41.7% in 2012, with an average annual increase of 2.5%±0.05% (Wald test for trend: *p*<0.001). The mean frequency of SMBG per day continuously increased from 2.8±1.4 per day in 1995 to 5.5±1.9 per day in 2012, with an average annual increase of 0.11±0.002 per day (Wald test for trend: *p*<0.001). A total of 6,517 events of severe hypoglycemia in 3,372 patients (9.0% of patients) and 1,169 events of hypoglycemic coma in 897 patients (2.4%) were documented of 155,240 medical visits.

### Temporal Trends of Severe Hypoglycemia and Coma

The unadjusted mean rates of severe hypoglycemia and hypoglycemic coma within the whole observation period between 1995 and 2012 were 20.07 per 100 patient-years (95% CI 19.59 to 20.57) and 3.60 per 100 patient-years (95% CI 3.40 to 3.81), respectively. Mean rates of severe hypoglycemia and coma declined from 42.28 per 100 patient-years (95% CI 33.56 to 53.27) and 13.51 per 100 patient-years (95% CI 8.98 to 20.33) in 1995, respectively, to 17.63 per 100 patient-years (95% CI 16.92 to 18.37) and 1.82 per 100 patient-years (95% CI 1.60 to 2.06) in 2012, respectively. From 1995 to 2012, there was a significant decrease in severe hypoglycemia and hypoglycemic coma, as calculated by regression analysis, with RR per 1 y of 0.98 (95% CI 0.97 to 0.99, Wald test: *p*<0.001) and 0.92 (95% CI 0.91 to 0.94, Wald test: *p*<0.001), respectively, after adjustment for sex, age, and diabetes duration. This corresponds to a reduction in severe hypoglycemia and coma of 2% and 8% per consecutive year, respectively. Results were similar after additionally adjusting for insulin treatment regimen, with RR per year of 0.99 (95% CI 0.98 to 0.995, Wald test: *p* = 0.002) and 0.92 (95% CI 0.91 to 0.94, Wald test: *p*<0.001), respectively.

### Association between Hypoglycemia and HbA1c

To study the link between HbA1c and severe hypoglycemia or coma over time, trends in RRs for the period 1995 to 2012 were calculated by regression analysis, adjusted for age, sex, and diabetes duration. From 1995 to 2012, the RR for severe hypoglycemia and hypoglycemic coma per 1% HbA1c decrease declined from 1.28 to 1.05 and from 1.39 to 1.01, respectively (Table 2), indicating that the inverse association between severe hypoglycemia or coma and HbA1c decreased during the observation period. From 2010 onward, the RR for hypoglycemic coma per 1% HbA1c decrease was no longer significant (Table 2). The RR for severe hypoglycemia and coma per 1% decrease in HbA1c dropped on average by 1.2% (95% CI 0.6% to 1.7%) and 1.9% (95% CI 0.8% to 2.9%) each year, respectively (RR 0.988, 95% CI 0.983 to 0.994, Wald test for trend: *p*<0.001, and RR 0.981, 95% CI 0.971 to 0.992, Wald test for trend: *p*<0.001, respectively). These results remained significant after additional adjustment for insulin treatment regimen (RR 0.989, 95% CI 0.984 to 0.995, Wald test for trend: *p*<0.001, and RR 0.981, 95% CI 0.972 to 0.992, Wald test for trend: *p*<0.001, respectively).

**Table 2:** HbA1c as predictor of severe hypoglycemia and hypoglycemic coma per year, expressed as relative risk per 1% HbA1c decrease.

Year	Severe Hypoglycemia				Hypoglycemic Coma			
	Model 1		Model 2		Model 1		Model 2	
	RR (95% CI)	*p*-Value	RR (95% CI)	*p*-Value	RR (95% CI)	*p*-Value	RR (95% CI)	*p*-Value
1995	1.28 (1.19–1.37)	<0.001	1.27 (1.19–1.36)	<0.001	1.39 (1.23–1.56)	<0.001	1.39 (1.23–1.56)	<0.001
1996	1.26 (1.18–1.35)	<0.001	1.26 (1.18–1.34)	<0.001	1.36 (1.22–1.52)	<0.001	1.36 (1.22–1.52)	<0.001
1997	1.25 (1.18–1.32)	<0.001	1.24 (1.17–1.32)	<0.001	1.34 (1.21–1.48)	<0.001	1.34 (1.21–1.48)	<0.001
1998	1.23 (1.17–1.30)	<0.001	1.23 (1.17–1.30)	<0.001	1.31 (1.20–1.44)	<0.001	1.31 (1.20–1.44)	<0.001
1999	1.22 (1.16–1.28)	<0.001	1.22 (1.16–1.28)	<0.001	1.29 (1.18–1.40)	<0.001	1.29 (1.19–1.40)	<0.001
2000	1.20 (1.15–1.26)	<0.001	1.20 (1.15–1.26)	<0.001	1.26 (1.17–1.36)	<0.001	1.27 (1.17–1.36)	<0.001
2001	1.19 (1.14–1.24)	<0.001	1.19 (1.14–1.24)	<0.001	1.24 (1.16–1.33)	<0.001	1.24 (1.16–1.33)	<0.001
2002	1.18 (1.13–1.22)	<0.001	1.18 (1.13–1.22)	<0.001	1.22 (1.14–1.29)	<0.001	1.22 (1.15–1.30)	<0.001
2003	1.16 (1.12–1.20)	<0.001	1.17 (1.13–1.21)	<0.001	1.19 (1.13–1.26)	<0.001	1.20 (1.13–1.27)	<0.001
2004	1.15 (1.11–1.18)	<0.001	1.15 (1.12–1.19)	<0.001	1.17 (1.11–1.24)	<0.001	1.17 (1.11–1.24)	<0.001
2005	1.14 (1.10–1.17)	<0.001	1.14 (1.11–1.17)	<0.001	1.15 (1.09–1.21)	<0.001	1.15 (1.09–1.21)	<0.001
2006	1.12 (1.09–1.15)	<0.001	1.13 (1.10–1.16)	<0.001	1.13 (1.07–1.19)	<0.001	1.13 (1.07–1.19)	<0.001
2007	1.11 (1.08–1.14)	<0.001	1.12 (1.08–1.15)	<0.001	1.11 (1.05–1.17)	<0.001	1.11 (1.05–1.17)	<0.001
2008	1.10 (1.06–1.13)	<0.001	1.10 (1.07–1.14)	<0.001	1.09 (1.03–1.15)	0.005	1.09 (1.03–1.15)	0.004
2009	1.08 (1.05–1.12)	<0.001	1.09 (1.06–1.13)	<0.001	1.07 (1.00–1.14)	0.047	1.07 (1.00–1.14)	0.040
2010	1.07 (1.03–1.11)	<0.001	1.08 (1.04–1.12)	<0.001	1.05 (0.98–1.12)	0.201	1.05 (0.98–1.12)	0.181
2011	1.06 (1.02–1.10)	<0.001	1.07 (1.03–1.11)	0.002	1.03 (0.95–1.11)	0.497	1.03 (0.95–1.11)	0.463
2012	1.05 (1.00–1.09)	0.050	1.06 (1.01–1.10)	0.016	1.01 (0.93–1.10)	0.856	1.01 (0.93–1.10)	0.816

RRs with 95% CIs derived from regression models with severe hypoglycemia or hypoglycemic coma as the dependent variable and using continuous terms for HbA1c and treatment year. Model 1 adjusted for HbA1c, treatment year, sex, age, and diabetes duration, and with a term for interaction between HbA1c and treatment year; model 2 additionally adjusted for insulin treatment regimen (1–3 or ≥4 insulin injection time points per day, or insulin pump therapy). *p*-Values from Wald tests refer to RRs. Wald test for trend in RR per 1% HbA1c decrease: *p*<0.001 for severe hypoglycemia and coma in models 1 and 2.

**Figure 1:**
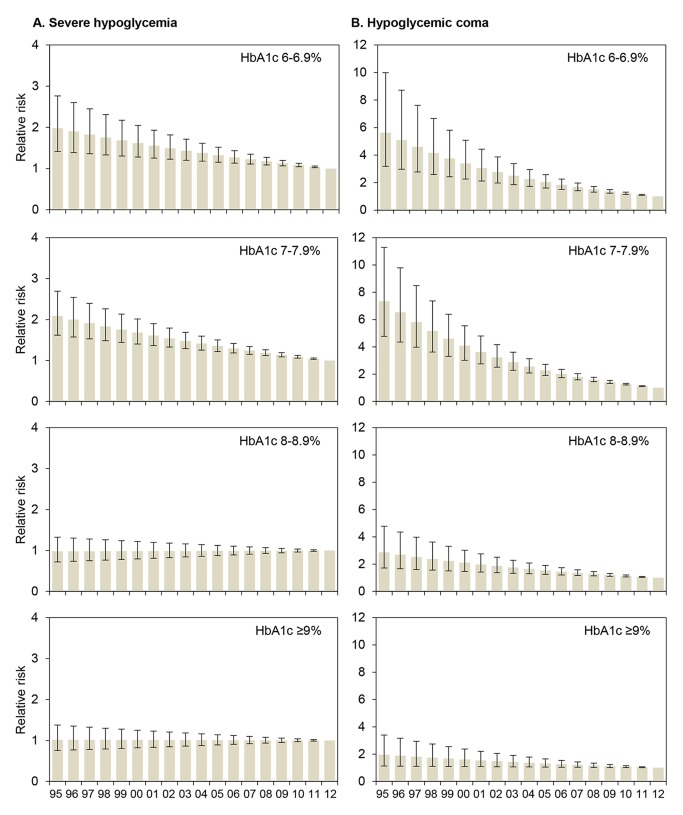
Relative risk for severe hypoglycemia and hypoglycemic coma by HbA1c category for each treatment year. RRs (with 95% CIs) are derived from regression analyses with severe hypoglycemia (A) or hypoglycemic coma (B) as the dependent variable, modeling treatment year (*x*-axis) as a continuous term and HbA1c as a categorical term and including a term for interaction between treatment year and HbA1c, adjusted for sex, age, and diabetes duration. 2012 is the reference year (RR 1.0). Conversion for HbA1c [mmol/mol]  =  (HbA1c [percent] − 2.15) ×10.929.

The RRs for severe hypoglycemia and hypoglycemic coma were also analyzed by HbA1c category for each year from 1995 to 2012 by regression analysis (Figure 1). The reduction of the RR for severe hypoglycemia (Figure 1A) was most prominent in patients with an HbA1c of 6.0%–6.9% (RR 0.96 each year) and HbA1c of 7.0%–7.9% (RR 0.96 each year), corresponding to a risk reduction of 4% per year and 50% during the whole observation period, while no changes were observed in patients with higher HbA1c levels (Table 3). Similarly, RR reduction of coma was strongest in patients with an HbA1c of 6.0%–6.9% (RR 0.90 each year) and 7.0%–7.9% (RR 0.89 each year) (Figure 1B; Table 3), corresponding to a risk reduction of 10% and 11% per year, respectively, and a risk reduction of 83% and 86% during the entire study period, respectively. These effects remained significant after additional adjustment for insulin treatment regimen (Table 3).

**Table 3:** Relative risk for severe hypoglycemia and hypoglycemic coma per year from 1995 to 2012 by HbA1c category.

HbA1c (Percent)	Severe Hypoglycemia		Hypoglycemic Coma	
	RR (95% CI) per Year	*p*-Value	RR (95% CI) per Year	*p*-Value
6.0%–6.9%	0.96 (0.94–0.98)	<0.001	0.90 (0.87–0.93)	<0.001
	0.97 (0.95–0.99)*	0.002	0.90 (0.87–0.94)*	<0.001
7.0%–7.9%	0.96 (0.94–0.97)	<0.001	0.89 (0.87–0.91)	<0.001
	0.96 (0.95–0.98)*	<0.001	0.89 (0.87–0.92)*	<0.001
8.0%–8.9%	1.00 (0.98–1.02)	0.886	0.94 (0.91–0.97)	<0.001
	1.01 (0.99–1.02)*	0.553	0.94 (0.91–0.97)*	<0.001
≥9.0%	1.00 (0.98–1.02)	0.907	0.96 (0.93–0.99)	0.018
	1.00 (0.99–1.02)*	0.713	0.96 (0.93–0.99)*	0.025

Results calculated from regression models with severe hypoglycemia or hypoglycemic coma as the dependent variable, adjusted for treatment year (continuous term), HbA1c category, sex, age, and diabetes duration, and with a term for interaction between HbA1c and treatment year. RRs with 95% CIs. *p*-Values from Wald tests are related to RR. Conversion for HbA1c [mmol/mol]  =  (HbA1c [percent] − 2.15) ×10.929.

*Additionally adjusted for insulin treatment regimen (1–3 or ≥4 insulin injection time points per day, or insulin pump therapy).

### Other Predictors of Severe Hypoglycemia and Coma

During the entire observation period from 1995 to 2012, older age was associated with moderately decreased risk of severe hypoglycemia (6% risk reduction per 1-y age increase; Table 4) and hypoglycemic coma (3% risk reduction per 1-y age increase; Table 4). Longer diabetes duration was a weak predictor of severe hypoglycemia (2%–3% risk increase per 1-y increase in diabetes duration) but not of coma risk (Table 4). Females had a 17% higher risk for severe hypoglycemia than males, while coma risk was similar in both sexes. There was no temporal trend per year in the association of age, diabetes duration, and sex with severe hypoglycemia and hypoglycemic coma, except a small annual 0.5%–0.6% increase in the risk for coma per 1-y age increase (Table 4).

**Table 4:** Association of age, diabetes duration, and sex with severe hypoglycemia and hypoglycemic coma for 1995–2012, expressed as relative risk and annual change in relative risk.

Risk Factor	Severe Hypoglycemia		Hypoglycemic Coma	
	RR (95% CI)	*p*-Value	RR (95% CI)	*p*-Value
**Age (per 1-y increase)**	0.944 (0.933–0.956)	<0.001	0.975 (0.955–0.996)	0.018
	0.938 (0.927–0.950)*	<0.001	0.970 (0.950–0.991)*	0.005
	1.001 (0.998–1.004)§	0.488	1.006 (1.001–1.010)§	0.009
	1.000 (0.997–1.002)* §	0.708	1.005 (1.001–1.010)* §	0.027
**Diabetes duration (per 1-y increase)**	1.023 (1.008–1.038)	<0.001	1.012 (0.988–1.037)	0.337
	1.028 (1.013–1.043)*	<0.001	1.013 (0.988–1.037)*	0.308
	0.999 (0.996–1.002)§	0.363	1.000 (0.995–1.004)§	0.863
	1.000 (0.997–1.003)* §	0.932	1.001 (0.996–1.006)* §	0.809
**Sex (female versus male)**	1.168 (1.072–1.272)	<0.001	0.952 (0.823–1.102)	0.511
	1.169 (1.073–1.273)*	<0.001	0.955 (0.825–1.105)*	0.533
	0.996 (0.979–1.013)§	0.646	0.981 (0.952–1.011)§	0.202
	0.998 (0.981–1.015)* §	0.797	0.985 (0.955–1.015)* §	0.315

Results derived from regression models with severe hypoglycemia or hypoglycemic coma as the dependent variable, adjusted for treatment year (continuous term), HbA1c (continuous term), sex, age, and diabetes duration, and with a term for interaction between treatment year and HbA1c. *p*-Values from Wald tests refer to RRs. For assessment of changes in the association of variables with severe hypoglycemia and hypoglycemic coma between treatment years, regression models additionally included terms for interactions between treatment year by age, diabetes duration, and sex.

*Additionally adjusted for insulin treatment regimen (1–3 or ≥4 insulin injection time points per day, or insulin pump therapy).

§ Estimate represents the average annual change in RR (corresponds to a term for interaction with treatment year in models; *p*-values from Wald tests).

## Discussion

To investigate the link between HbA1c and the risk of hypoglycemia, we conducted an observational study in a prospective cohort of 37,539 young patients with type 1 diabetes treated between 1995 and 2012 in Germany and Austria. Our major finding is that the RR for severe hypoglycemia and coma per 1% HbA1c decrease dropped by 1.2% and 1.9%, respectively, each year. The strong association between severe hypoglycemia and low HbA1c levels observed in 1995 (RR per 1% HbA1c decrease 1.28) has markedly decreased in the recent decade until 2012 (RR per 1% HbA1c decrease 1.05). Thus, HbA1c has become a minor predictor for severe hypoglycemia in young patients with type 1 diabetes. This effect was caused by a substantial reduction of hypoglycemia risk from 1995 to 2012 exclusively in patients with HbA1c levels of 6.0%–6.9% and 7.0%–7.9% (50% risk reduction). Similarly, the risk of hypoglycemic coma decreased preferentially in patients in low HbA1c categories (83% and 86% risk reduction, respectively, for HbA1c levels of 6.0%–6.9% and 7.0%–7.9%).

Our finding of a significant decline in hypoglycemia and coma rates in patients with low HbA1c during recent years is consistent with previous observations in smaller population-based studies [16]. The overall rate of severe hypoglycemia in our study was similar to that of another population-based investigation and a recent randomized controlled trial (17.9 per 100 patient-years) [4],[28], but lower than in studies from tertiary diabetes centers (29.4 to 55.4 per 100 patient-years) [19],[21]. The rate of hypoglycemic coma in our population was lower than that described in other studies (8 to 26 per 100 patient-years) [5]–[7],[11],[17]–[19],[21],[29] but similar to that in a Finnish population (3.1 to 3.6 per 100 patient-years) [30]. It may be assumed that differences in study design, patient selection, and case definition have contributed to the variability of hypoglycemic event rates observed in these studies.

Hypoglycemia risk may be affected by a variety of treatment-related factors, including type of insulin, route of insulin administration, and SMBG frequency [31],[32]. In our cohort, the use of short-acting insulin analogs, long-acting insulin analogs, and insulin pumps increased during the observation period from 1995 to 2012. Clinical trials have shown that the use of insulin analogs [31],[33],[34] or pumps [29],[31] may be associated with a reduced risk of nocturnal or daytime hypoglycemia in patients with type 1 diabetes, while achieving good metabolic control. It is tempting to speculate about a link between trends in insulin therapy and hypoglycemia reduction, but our study was not designed to investigate effects of different treatment modalities on hypoglycemia risk. In addition, although we found a significant temporal increase in SMBG frequency, no conclusions about the influence of SMBG frequency on hypoglycemia risk should be drawn from our data.

Other predictors of severe hypoglycemia identified in this study were younger age, longer diabetes duration, and female sex, in line with previous studies [4]–[7],[11],[18],[19],[28]. However, the RR of hypoglycemia attributable to each of these variables was small, and in contrast to HbA1c-related risk, no temporal changes were identified in the observation period of 18 y. Our work is limited by the fact that diabetes education and physical activity was not addressed, both relevant to hypoglycemia risk [12],[30],[35] but difficult to assess quantitatively in a large population.

A strength of this study is its large, population-based multicenter database with prospective documentation of “real-life” diabetes care, suitable for analyzing temporal trends over almost two decades [8]. The study was performed in more than 37,000 children, adolescents, and young adults with type 1 diabetes treated in 349 diabetes clinics in Germany and Austria, with an estimated nationwide capture rate of >80%. Patient selection and data acquisition remained unchanged during the entire study period, reducing potential bias. Taken together, our results are likely representative of young type 1 diabetes patients of European (but not other) descent with a diabetes duration of two or more years.

Our data may have potential implications for the clinical management of patients with type 1 diabetes. Lower HbA1c and hypoglycemia have been traditionally viewed as two sides of one coin, a notion that is supported by the data from the early years of observation in our study, as well as by other reports [4]–[6],[11],[15],[26]. Accordingly, hypoglycemia has been considered a barrier preventing the benefits of near-normal glycemic control [13],[14],[26],[36]. Our observation that the negative association between HbA1c and hypoglycemia risk has decreased indicates that strict glycemic control has become safer in recent years. Consequently, patients and health care professionals may be reassured that improved glycemic control—and thereby risk reduction for chronic diabetes complications—is achievable in young patients with type 1 diabetes without inherently increasing the risk of severe hypoglycemia. Longitudinal studies have highlighted the effect of intensive diabetes counseling on hypoglycemia prevention, resulting in a reduction of severe hypoglycemia rates of 32% [35] to 62% [12] 1 y after a structured treatment course. Intensive and repeated diabetes education for patients and, if required, their families should therefore remain an integral part of type 1 diabetes treatment strategies [12],[30]. As the contribution of diabetes education and other individual modifiable factors to hypoglycemia reduction remains undefined in this observational study, further systematic investigation will be required.

In conclusion, the results from this study show that the former association between the risk of severe hypoglycemia and low HbA1c levels has largely decreased during the last decade in young patients with type 1 diabetes. Thus, in Germany and Austria, low HbA1c is no longer a strong predictor of severe hypoglycemia in young patients with type 1 diabetes, reducing the barriers to achieving and maintaining near-normal glycemic control.

## Supporting Information

Text S1 **Participating DPV centers.**
(DOCX)

Text S2 **STROBE checklist.**
(DOC)

## Abbreviations

BMI: body mass index
DCCT: Diabetes Control and Complications Trial
HbA1c: hemoglobin A1c
Q1: quartile 1
Q3: quartile 3
RR: relative risk
SD: standard deviation
SMBG: self-monitoring of blood glucose
