# Supplementary material for: Hemoglobin A1c Levels and Risk of Severe Hypoglycemia in Children and Young Adults with Type 1 Diabetes from Germany and Austria: A Trend Analysis in a Cohort of 37,539 Patients between 1995 and 2012
Source: PLoS Med. 2014 Oct 7;11(10):e1001742. doi: 10.1371/journal.pmed.1001742 (PMC4188517; doi:10.1371/journal.pmed.1001742)
Supplement: Text S1 — Participating DPV centers. (DOCX) [file pmed.1001742.s001.docx]

**Participating DPV centers**

Aachen - Innere RWTH, Aachen - Uni-Kinderklinik RWTH, Aalen Kinderklinik, Ahlen St. Franziskus Kinderklinik, Altötting Zentrum Inn-Salzach, Altötting-Burghausen Innere Medizin, Amstetten Klinikum Mostviertel Kinderklinik, Arnsberg-Hüsten Karolinenhosp. Kinderabteilung, Asbach Kamillus-Klinik Innere, Aue Helios Kinderklink, Augsburg Innere, Augsburg Kinderklinik Zentralklinikum, Aurich Kinderklinik, Bad Aibling Internist. Praxis, Bad Driburg / Bad Hermannsborn Innere, Bad Hersfeld Kinderklinik, Bad Kösen Kinder-Rehaklinik, Bad Lauterberg Diabeteszentrum Innere, Bad Mergentheim - Diabetesfachklinik, Bad Mergentheim - Gemeinschaftspraxis DM-dorf Althausen, Bad Oeynhausen Herz-und Diabeteszentrum NRW, Bad Orb Spessart Klinik, Bad Orb Spessart Klinik Reha, Bad Reichenhall Kreisklinik Innere Med., Bad Salzungen Kinderklinik, Bad Säckingen Hochrheinklinik Innere, Bad Waldsee Kinderarztpraxis, Bautzen Oberlausitz KK, Bayreuth Innere Medizin, Berchtesgaden CJD, Berchtesgaden MVZ Innere Med, Berlin DRK-Kliniken, Berlin Endokrinologikum, Berlin Evang. Krankenhaus Königin Elisabeth, Berlin Klinik St. Hedwig Innere, Berlin Lichtenberg - Kinderklinik, Berlin St. Josephskrankenhaus Innere, Berlin Virchow-Kinderklinik, Berlin Vivantes Hellersdorf Innere, Bielefeld Kinderklinik Gilead, Bocholt Kinderklinik, Bochum Universitätskinderklinik St. Josef, Bonn Uni-Kinderklinik, Bottrop Kinderklinik, Bottrop Knappschaftskrankenhaus Innere, Braunschweig Kinderarztpraxis, Bremen - Kinderklinik Nord, Bremen - Mitte Innere, Bremen Prof. Hess Kinderklinik, Bremerhaven Kinderklinik, Böblingen Kinderklinik, Celle Klinik für Kinder- und Jugendmedizin, Chemnitz Kinderklinik, Chemnitz-Hartmannsdorf Innere Medizin - DIAKOMED-1, Coesfeld Kinderklinik, Coesfeld/Dülmen Innere Med., Darmstadt Innere Medizin, Darmstadt Kinderklinik Prinz. Margaret, Datteln Vestische Kinderklinik, Deggendorf Kinderarztpraxis, Deggendorf Kinderklinik, Deggendorf Medizinische Klinik II, Delmenhorst Kinderklinik, Dessau Kinderklinik, Detmold Kinderklinik, Dornbirn Kinderklinik, Dortmund Kinderklinik, Dortmund Knappschaftskrankenhaus Innere, Dortmund Medizinische Kliniken Nord, Dortmund-Hombruch Marienhospital, Dortmund-St. Josefshospital Innere, Dresden Neustadt Kinderklinik, Dresden Uni-Kinderklinik, Duisburg Evang. und Johanniter Krhs, Innere, Duisburg Kinderklinik, Duisburg Malteser St. Anna Innere, Duisburg Malteser St. Johannes, Duisburg-Huckingen, Duisburg-St.Johannes Helios, Düren-Birkesdorf Kinderklinik, Düsseldorf Uni-Kinderklinik, Eberswalde Klinikum Barnim Werner Forßmann - Innere, Erfurt Kinderklinik, Erlangen Uni Innere Medizin, Erlangen Uni-Kinderklinik, Essen Diabetes-Schwerpunktpraxis, Essen Elisabeth Kinderklinik, Essen Uni-Kinderklinik, Esslingen Klinik für Kinder und Jugendliche, Eutin Kinderklinik, Feldkirch Kinderklinik, Forchheim Diabeteszentrum SPP, Frankenthal Kinderarztpraxis, Frankfurt Diabeteszentrum Rhein-Main-Erwachsenendiabetologie (Bürgerhospital), Frankfurt Uni-Kinderklinik, Frankfurt Uni-Klinik Innere, Freiburg Kinder-MVZ, Freiburg St. Josef Kinderklinik, Freiburg Uni Innere, Freiburg Uni-Kinderklinik, Friedberg Innere Klinik, Friedrichshafen Kinderklinik, Fulda Innere Medizin, Fulda Kinderklinik, Fürth Kinderklinik, Gaissach Fachklinik der Deutschen Rentenversicherung Bayern Süd, Garmisch-Partenkirchen Kinderklinik, Geislingen Klinik Helfenstein Innere, Gelnhausen Innere, Gelnhausen Kinderklinik, Gelsenkirchen Kinderklinik Marienhospital, Gera Kinderklinik, Gießen Ev. Krankenhaus Mittelhessen, Gießen Uni-Kinderklinik, Graz Universitäts-Kinderklinik, Göppingen Kinderklinik am Eichert, Görlitz Städtische Kinderklinik, Göttingen Uni-Kinderklinik, Güstrow Innere, Hachenburg Kinderpraxis, Hagen Kinderklinik, Halle Uni-Kinderklinik, Halle-Dölau Städtische Kinderklinik, Hamburg Altonaer Kinderklinik, Hamburg Endokrinologikum, Hamburg Kinderklinik Wilhelmstift, Hamburg-Nord Kinder-MVZ, Hameln Kinderklinik, Hamm Kinderklinik, Hanau Kinderklinik, Hanau St. Vincenz - Innere, Hannover Henriettenstift - Innere, Hannover Kinderklinik MHH, Hannover Kinderklinik auf der Bult, Haren Kinderarztpraxis, Heide Kinderklinik, Heidelberg Uni-Kinderklinik, Heidenheim Kinderklinik, Heilbronn Innere Klinik, Heilbronn Kinderklinik, Herdecke Kinderklinik, Herford Innere Med I, Herford Kinderarztpraxis, Herford Klinikum Kinder & Jugendliche, Heringsdorf Inselklinik, Hermeskeil Kinderpraxis, Herne Evan. Krankenhaus Innere, Herten St. Elisabeth Innere Medizin, Hildesheim Kinderarztpraxis, Hildesheim Klinikum Kinderklinik, Hinrichsegen-Bruckmühl Diabetikerjugendhaus, Hof Kinderklinik, Homburg Uni-Kinderklinik Saarland, Idar Oberstein Innere, Ingolstadt Klinikum Innere, Innsbruck Universitätskinderklinik, Iselsberg - Rehazentrum Ederhof, Iserlohn Innere Medizin, Itzehoe Kinderklinik, Jena Uni-Kinderklinik, Kaiserslautern Kinderarztpraxis, Kaiserslautern-Westpfalzklinikum Kinderklinik, Karlsburg Klinik für Diabetes & Stoffwechsel, Karlsruhe Städtische Kinderklinik, Kassel Klinikum Kinder- und Jugendmedizin, Kassel Städtische Kinderklinik, Kaufbeuren Innere Medizin, Kempen Heilig Geist - Innere, Kiel Städtische Kinderklinik, Kiel Universitäts-Kinderklinik, Kirchen DRK Klinikum Westerwald Kinderklinik, Kirchheim-Nürtingen Innere, Kleve Innere Medizin, Klinikum Hildesheim GmbH Innere, Koblenz Kemperhof 1. Med. Klinik, Koblenz Kinderklinik Kemperhof, Konstanz Innere Klinik, Konstanz Kinderklinik, Krefeld Innere Klinik, Krefeld Kinderklinik, Krefeld-Uerdingen St. Josef Innere, Kreischa-Zscheckwitz, Klinik Bavaria, Köln Kinderklinik Amsterdamerstrasse, Köln Uni-Kinderklinik, Landshut Kinderklink, Lappersdorf Kinderarztpraxis, Leipzig Uni-Kinderklinik, Leoben LKH Kinderklinik, Leverkusen Kinderklinik, Lienz BKH Pädiatrie, Limburg Innere Medizin, Lindenfels Luisenkrankenhaus Innere, Lingen Kinderklinik St. Bonifatius, Linz Krankenhaus Barmherzige Schwestern Kardiologie Abt. Int. II, Linz Krankenhaus der Barmherzigen Schwestern Kinderklinik, Linz Landes-Kinderklinik, Lippstadt Evangelische Kinderklinik, Ludwigsburg Innere Medizin, Ludwigsburg Kinderklinik, Ludwigshafen Kinderklinik St.Anna-Stift, Ludwigshafen diabetol. SPP, Lübeck Uni-Kinderklinik, Lübeck Uni-Klinik Innere Medizin, Lüdenscheid Hilfswerk Kinder & Jugendliche, Lüdenscheid Märkische Kliniken - Kinder & Jugendmedizin, Magdeburg Städtisches Klinikum Innere, Magdeburg Uni-Kinderklinik, Mainz Uni-Kinderklinik, Mannheim Uni-Kinderklinik, Mannheim Uniklinik Innere Medizin, Marburg - UKGM Endokrinologie & Diabetes, Marburg Uni-Kinderklinik, Marktredwitz Innere Medizin, Mechernich Kinderklinik, Memmingen Kinderklinik, Merzig Kinderklinik, Minden Kinderklinik, Moers - St. Josefskrankenhaus Innere, Moers Kinderklinik, Murnau am Staffelsee - diabetol. SPP, Mutterstadt Kinderarztpraxis, Mödling Kinderklinik, Mönchengladbach Kinderklinik Rheydt Elisabethkrankenhaus, Mühlacker Enzkreiskliniken Innere, Mühldorf Gemeinschaftspraxis, München 3. Orden Kinderklinik, München Diabetes-Zentrum Süd, München Kinderarztpraxis diabet. SPP, München Schwerpunktpraxis Evers, München von Haunersche Kinderklinik, München-Gauting Kinderarztzentrum, München-Harlaching Kinderklinik, München-Schwabing Kinderklinik, Münster Herz Jesu Innere, Münster St. Franziskus Kinderklinik, Münster Uni-Kinderklinik, Münster pädiat. Schwerpunktpraxis, Nauen Havellandklinik, Neuburg Kinderklinik, Neumarkt Innere, Neunkirchen Innere Medizin, Neunkirchen Marienhausklinik Kohlhof Kinderklinik, Neuss Lukaskrankenhaus Kinderklinik, Neuwied Kinderklinik Elisabeth, Neuwied Marienhaus Klinikum St. Elisabeth Innere, Nürnberg Cnopfsche Kinderklinik, Nürnberg Zentrum f Neugeb., Kinder & Jugendl., Oberhausen Innere, Oberhausen Kinderklinik, Oberhausen Kinderpraxis, Oberhausen St.Clemens Hospitale Sterkrade, Offenbach/Main Innere Medizin, Offenbach/Main Kinderklinik, Offenburg Kinderklinik, Oldenburg Kinderklinik, Oldenburg Schwerpunktpraxis, Osnabrück Christliches Kinderhospital, Osterkappeln Innere, Ottobeuren Kreiskrankenhaus, Oy-Mittelberg Hochgebirgsklinik Kinder-Reha, Paderborn St. Vincenz Kinderklinik, Papenburg Marienkrankenhaus Kinderklinik, Passau Kinderarztpraxis, Passau Kinderklinik, Pforzheim Kinderklinik, Pfullendorf Innere Medizin, Pirmasens Städtisches Krankenhaus Innere, Plauen Vogtlandklinikum, Prenzlau Krankenhaus Innere, Rastatt Gemeinschaftspraxis, Rastatt Kreiskrankenhaus Innere, Ravensburg Kinderklink St. Nikolaus, Recklinghausen Dialysezentrum Innere, Regensburg Kinderklinik St. Hedwig, Remscheid Kinderklinik, Rendsburg Kinderklinik, Reutlingen Kinderarztpraxis, Reutlingen Kinderklinik, Reutlingen Klinikum Steinenberg Innere, Rheine Mathiasspital Kinderklinik, Rosenheim Innere Medizin, Rosenheim Kinderklinik, Rosenheim Schwerpunktpraxis, Rostock Uni-Kinderklinik, Rostock Universität Innere Medizin, Rotenburg/Wümme Kinderklinik, Rüsselsheim Kinderklinik, Saaldorf-Surheim Diabetespraxis, Saalfeld Thüringenklinik Kinderklinik, Saarbrücken Kinderklinik Winterberg, Saarlouis Kinderklinik, Salzburg Kinderklinik, Scheidegg Prinzregent Luitpold, Scheidegg Reha-Kinderklinik Maximilian, Schw. Gmünd Stauferklinik Kinderklinik, Schweinfurt Kinderklinik, Schwerin Innere Medizin, Schwerin Kinderklinik, Schwäbisch Hall Diakonie Kinderklinik, Siegen Kinderklinik, Singen - Hegauklinik Kinderklinik, Sinsheim Innere, Spaichingen Innere, St. Augustin Kinderklinik, St. Pölten Kinderklinik, Stade Kinderklinik, Stolberg Kinderklinik, Stuttgart Olgahospital Kinderklinik, Suhl Kinderklinik, Sylt Rehaklinik, Tettnang Innere Medizin, Traunstein diabetol. Schwerpunktpraxis, Trier Kinderklinik der Borromäerinnen, Trostberg Innere, Tübingen Uni-Kinderklinik, Ulm Endokrinologikum, Ulm Schwerpunktpraxis Bahnhofsplatz, Ulm Uni-Kinderklinik, Vechta Kinderklinik, Viersen Kinderkrankenhaus St. Nikolaus, Villach Kinderklinik, Villingen-Schwenningen SPP, Villingen-Schwenningen Schwarzwald-Baar-Klinikum Innere, Waiblingen Kinderklinik, Waldshut Kinderpraxis, Waldshut-Tiengen Kinderpraxis Biberbau, Waren-Müritz Kinderklinik, Weiden Kinderklinik, Weingarten Kinderarztpraxis, Weisswasser Kreiskrankenhaus, Wels Klinikum, Wernberg-Köblitz SPP, Wetzlar Schwerpunkt-Praxis, Wetzlar/Braunfels Innere, Wien Preyersches Kinderspital, Wien Rudolfstiftung, Wien SMZ Ost Donauspital, Wien Uni-Kinderklinik, Wiesbaden Horst-Schmidt-Kinderkliniken, Wiesbaden Kinderklinik DKD, Wilhelmshaven Reinhard-Nieter-Kinderklinik, Wilhelmshaven St. Willehad Innere, Wittenberg Innere Medizin, Wittenberg Kinderklinik, Wolgast Innere Medizin, Worms - Weierhof, Worms Kinderklinik, Wuppertal Kinderklinik.
